# Social media use, social identification and cross-cultural adaptation of international students: A longitudinal examination

**DOI:** 10.3389/fpsyg.2022.1013375

**Published:** 2022-11-10

**Authors:** Leonor Gaitán-Aguilar, Joep Hofhuis, Kinga Bierwiaczonek, Carmen Carmona

**Affiliations:** ^1^Erasmus Research Center for Media, Communication, and Culture (ERMeCC), Erasmus University Rotterdam, Rotterdam, Netherlands; ^2^Department of Psychology, University of Oslo, Oslo, Norway; ^3^Department of Research Methods and Diagnosis in Education, University of Valencia, Valencia, Spain

**Keywords:** acculturation, cross-cultural adaptation, social identification, social media, international students, longitudinal design

## Abstract

The mobility experience is an important life event for international students, and achieving successful psychological and sociocultural adaptation is crucial for this experience to be positive. Through a three-wave longitudinal study among international students enrolled at universities in Spain, Portugal, and Poland (*n* = 233), we examined the relationships between social media use, social identification, and (sociocultural and psychological) adaptation across time. Results of cross lagged panel modeling (CLPM) showed that social media contact with home nationals predicted greater identification with this group. Social media contact with host country nationals predicted poorer adaptation. Social media contact with other international students did not show any effects, while identification with this group predicted better adaptation. Our results point to the dynamic nature of the adaptation process, showing that the role of social media use and identification targeted at different social groups may play different roles than was previously found in cross-sectional research.

## Introduction

The number of international students enrolled in tertiary education, which increased from 2 million in 1998 to 6.1 million in 2019, continues rising due to educational policies that encourage mobility and intercultural skills in the current globalized market (OECD, 2021). The mobility experience can be an exciting opportunity for this group. However, adapting to a host country can also be stressful, and result in homesickness, feelings of isolation, anxiety and depression (Brunsting et al., 2018). Furthermore, challenges arise that range from language barriers and learning how to adjust to the new context, to questioning (new) identities (Gautam et al., 2016). Although these challenges are experienced differently by different individuals (see Demes and Geeraert, 2015), scholars and practitioners have been in search of protective factors that may benefit the overall population of international students, to ensure a positive educational experience (Kuo, 2014; Yu et al., 2014).

One major protective factor that has emerged from the literature are social ties (Kuo, 2014). Social ties facilitate cross-cultural adaptation (Brunsting et al., 2018) across different contexts and populations (Bierwiaczonek and Waldzus, 2016) as they provide international students with necessary resources to cope with the challenges of international transitions, and to develop a sense of belonging in a new country (Ward et al., 2001). While the major theoretical models of acculturation (e.g., Berry, 1997; Ward et al., 2001) mainly focus on host and home culture ties, international students’ environment reflects complex intergroup dynamics with multi-national and multicultural social networks that can influence their acculturation process (Hendrickson et al., 2011; Pekerti et al., 2020; Szabó et al., 2020). Thus, in order to contribute to a more comprehensive approach to international student adaptation, the first aim of the present study is to explore the role that social identification with the group of other international students has in cross-cultural adaptation, in addition to the social identification with home- and host national groups.

Furthermore, recent technological advancements have opened up new opportunities for social interaction while abroad (Wen, 2020). Especially the development of social media networks has had a profound impact on the experiences of international students, for example for offering additional social support (Hofhuis et al., 2019; Li and Peng, 2019; Pang, 2020) and expanding one’s social network during and after international transitions (Croucher and Rahmani, 2015). The majority of studies in this area focus on the role that social media play in keeping communication with family and friends in the home country and with host nationals in the host country (e.g., Cemalcilar et al., 2005; Park et al., 2014; Park and Noh, 2018; Hofhuis et al., 2019; Pang, 2020) again overlooking other available social groups, such as fellow foreigners. Thus, the second aim of the current study is to analyze the effect of social media on the cross-cultural adaptation of international students considering the different available social groups in the host country.

Since sojourner adaptation is a dynamic process evolving over time, scholars have called for more longitudinal research in studying this phenomenon (Smith and Khawaja, 2011; Ward and Geeraert, 2016; Brunsting et al., 2018; Shu et al., 2020; Bierwiaczonek and Kunst, 2021; Kunst, 2021). Longitudinal designs give insights into the temporal order and direction of the relationship among variables (Bryman, 2012), critical for assessing factors that change over time during the acculturation process (Ward et al., 1998; Hechanova-Alampay et al., 2002; Demes and Geeraert, 2015; Bierwiaczonek and Kunst, 2021). Therefore, they contribute to a better understanding of theoretical assumptions regarding the dynamic relationship between different predictor variables and the adaptation of migrants and sojourners (Ramos et al., 2016; Ward and Szabó, 2019). Pioneering longitudinal research in this field has examined social media use (e.g., Hendrickson and Rosen, 2017; Hu et al., 2018; Billedo et al., 2019, 2020), social identification (e.g., Cemalcilar and Falbo, 2008; Geeraert and Demoulin, 2013; Hirai et al., 2015; Waßmuth and Edinger-Schons, 2018) and acculturation (e.g., Serrano-Sánchez et al., 2021) among the international student population. However, with some exceptions (Hirai et al., 2015; Hendrickson and Rosen, 2017), previous longitudinal research has focused on host and home country groups only. Thus, the third aim of the present study is to examine social media use and social identification with the three main groups available in the host society and their longitudinal effects on cross-cultural adaptation.

In sum, the present study departs from the notion that international students’ acculturation processes, operationalized through identification with different groups of individuals in the host society, can have a positive or negative impact on adaptation over time. Furthermore, the development and popularization of social media has opened up new ways in which sojourners can seek contact and exchange experiences with others, which may in turn affect their social identities. This study is one of the first to examine how these processes affect each other across time, using a longitudinal approach.

## Cross-cultural adaptation

The consensus among scholars is that cross-cultural adaptation has two dynamic dimensions that develop over time (Demes and Geeraert, 2015; Hirai et al., 2015): psychological and sociocultural adaptation (Searle and Ward, 1990; Ward and Searle, 1991; Ward and Kennedy, 1994; Ward et al., 2001). Psychological adaptation refers to the general well-being of the acculturating individual. It is usually examined using a stress and coping approach; sojourners learn to deal with the stress of living abroad using different resources (Ward et al., 2001; Ward and Szabó, 2019). Accordingly, previous studies have identified numerous stressors influencing psychological adaptation, including language barriers, perceived discrimination, and unfamiliar social norms (Smith and Khawaja, 2011), but also social coping resources, such as social ties and social support (Smith and Khawaja, 2011; Bierwiaczonek and Waldzus, 2016).

Sociocultural adaptation, based on the culture learning approach and reflecting a learning curve over time, refers to sojourners’ ability to manage everyday tasks in the new culture. This entails identifying and internalizing the specific norms, values, and behaviors of the host society, and developing practical skills to navigate the new culture (Ward et al., 2001; Wilson et al., 2013; Ward and Szabó, 2019). Among the factors affecting sociocultural adaptation are previous cross-cultural experiences, language proficiency, contact with host nationals (Wilson et al., 2013), length of stay in host country, discrimination, belonging and social interactions (Brunsting et al., 2018; Vakkai et al., 2020). Social ties are relevant for both dimensions of adaptation, although for different reasons: they help with psychological adaptation by enhancing coping, and with sociocultural adaptation, by facilitating culture learning (Bierwiaczonek and Waldzus, 2016). Because both dimensions are dynamic (Demes and Geeraert, 2015; Hirai et al., 2015), we argue that the relationship between social ties and adaptation can be best understood across time.

## Social identification and cross-cultural adaptation

One of the major predictors of both psychological and sociocultural adaptation is the sojourner’s acculturation process (Ward and Kus, 2012). Acculturation is defined through beliefs, attitudes, values, and identities that migrants and sojourners develop as a result of having intercultural contact (Berry et al., 2006). According to Vuong and Napier (2015), this development occurs as individuals evaluate (new) cultural values, that if found central to their self-concept are retained, and if not, discarded. Acknowledging the role of social ties in this process, the current study approaches acculturation from a social identity perspective (Tajfel and Turner, 1986), which proposes group belongingness as a cornerstone of individuals’ self-concept that guides beliefs and behaviors. Thus, we zoom in on identification processes that international students undergo as a result of their interactions with different social groups while studying abroad, and analyze their effect on cross-cultural adaptation (Ward et al., 2001).

Encounters with host nationals who speak a different language and act according to unfamiliar social norms, may make this intergroup context salient, accentuate the foreignness of international students, and boost a feeling of belonging elsewhere (Bierwiaczonek et al., 2017). Yet, research shows that feelings of identification and belonging to groups in one’s current environment are crucial to maintaining healthy well-being, self-esteem, and even somatic health (Greenaway et al., 2015) in the face of challenges such as intercultural transitions (e.g., Taušová et al., 2019). Therefore, adaptation may be easier if international students have a group they feel they belong to and identify with. Intergroup research (e.g., the rejection-identification model; Branscombe et al., 1999) showed that members of minorities increase their identification with their minority in-group as a way to cope with adversities coming from the majority (e.g., prejudice). This holds true in the case of international students; identification functions as a coping mechanism that can help them face acculturative stressors (Schmitt et al., 2003; Ramos et al., 2013).

However, research also shows that identifying with some groups helps adaptation, while identifying with others can hinder it (Ramos et al., 2013; Gomes et al., 2014; Bierwiaczonek et al., 2017; Beech, 2018). Therefore, it is crucial to account for the different identity groups available to international students in order to better grasp the role of identification processes in cross-cultural adaptation (Verkuyten et al., 2019). This study includes the three main groups that international students interact with in the host country (Bochner et al., 1977; from here onwards ‘target groups’): people from their home country that live in their host country (local home nationals), people from their host country (host nationals), and other international students.

### Identification with local home nationals

While studies showed the overall negative effect of home country identification, especially for sociocultural adaptation (Ward and Searle, 1991; Berry et al., 2006), few differentiated between identification with home nationals in the home country and home nationals in the host country (Szabó et al., 2020). Yet, this latter group may offer more benefits. By sharing the same contextual experience in the host country, this group can become a major source of social support, enhance a greater sense of belonging, promote home culture values, and share cultural skills that are relevant in the host society (Ward et al., 2010). Also, losing one’s home country networks as a consequence of moving abroad may hinder adaptation, while having local home national networks available (virtually or face to face) may enhance international students’ well-being and prevent isolation (Sawir et al., 2008). Thus, we expect that high identification with local home nationals is associated with better psychological (Hypothesis 1a) and sociocultural adaptation (Hypothesis 1b) over time.

### Identification with host nationals

While host nationals might not be the most accessible or preferred identity group for international students (e.g., Ramos et al., 2016), if available, they can become a valuable coping and learning resource (Berry et al., 2006). In acculturation studies, identification with this group tends to predict positive adaptation outcomes (Sam and Berry, 2010). Sojourners with higher host-national identification seem to experience better sociocultural adaptation (e.g., Ward and Kennedy, 1994; Cemalcilar et al., 2005) and report higher well-being (e.g., Waßmuth and Edinger-Schons, 2018; Serrano-Sánchez et al., 2021). Thus, we expect that high identification with host nationals is associated with better psychological (Hypothesis 2a) and sociocultural adaptation (Hypothesis 2b) over time.

### Identification with other international students

Possibly because study abroad is often a short-term experience, international students tend to be less involved with host nationals (Ward et al., 2001), and more in contact with other international sojourners who are in a similar situation (e.g., Sigalas, 2010; Fincher and Shaw, 2011; Williams and Johnson, 2011; Schartner, 2015). Fellow international students share the same experience of being away from home and face similar acculturative and academic challenges, which may bring them closer together and allow for a stronger social identification. Only a limited number of studies have been conducted on the effects of this particular social identity. The general conclusion seems to be that social ties and support from international students are positively related to more adaptive acculturation strategies (Kashima and Loh, 2006; Sullivan and Kashubeck-West, 2015; Cao et al., 2018), and that social identification with this group may mitigate the negative effects of acculturative stressors, such as prejudice and perceived discrimination (e.g., Schmitt et al., 2003; Bierwiaczonek et al., 2017). In line with these findings, we expect that social identification with international students has a positive impact on both psychological (Hypothesis 3a) and sociocultural adaptation (Hypothesis 3b).

## The impact of social media use

Media channels are important tools that sojourners have at their disposal during the adaptation process (Moon and Park, 2007; Alencar and Deuze, 2017). This may be especially true for social media, which allow for rapid and synchronous interpersonal communication across the globe facilitating contact with diverse networks worldwide (Boyd and Ellison, 2007; Forbush and Foucault-Welles, 2016). Social media contribute to the acculturation process by providing access to social support and relevant information throughout the international experience (Park et al., 2014; Billedo et al., 2019; Hofhuis et al., 2019; Pang, 2020). Given that today’s international students are among the first native users of the internet, social media represent main communication and informational resources that are used daily with diverse groups in both personal and professional settings (Wang et al., 2012; Tkalac Verčič and Verčič, 2013).

Earlier research has found that social media use with different groups (e.g., host or home nationals) may increase identification with those groups (McKelvy and Chatterjee, 2017). For instance, Cemalcilar et al. (2005) found that international students who communicated *via* social media with other co-nationals increased their identification with this group; similar results have also been found for the group of host nationals (e.g., Sawyer and Chen, 2012). Not all information on social media is relevant for the acculturation experience of international students, and different factors can mediate the effects of social media use (e.g., language; Li and Tsai, 2015). Thus, international students might evaluate and filter the information and values shared through social media, and if deemed useful, incorporate them into their self-concept (see *mindsponge* mechanism; Vuong and Napier, 2015).

**Figure 1:**
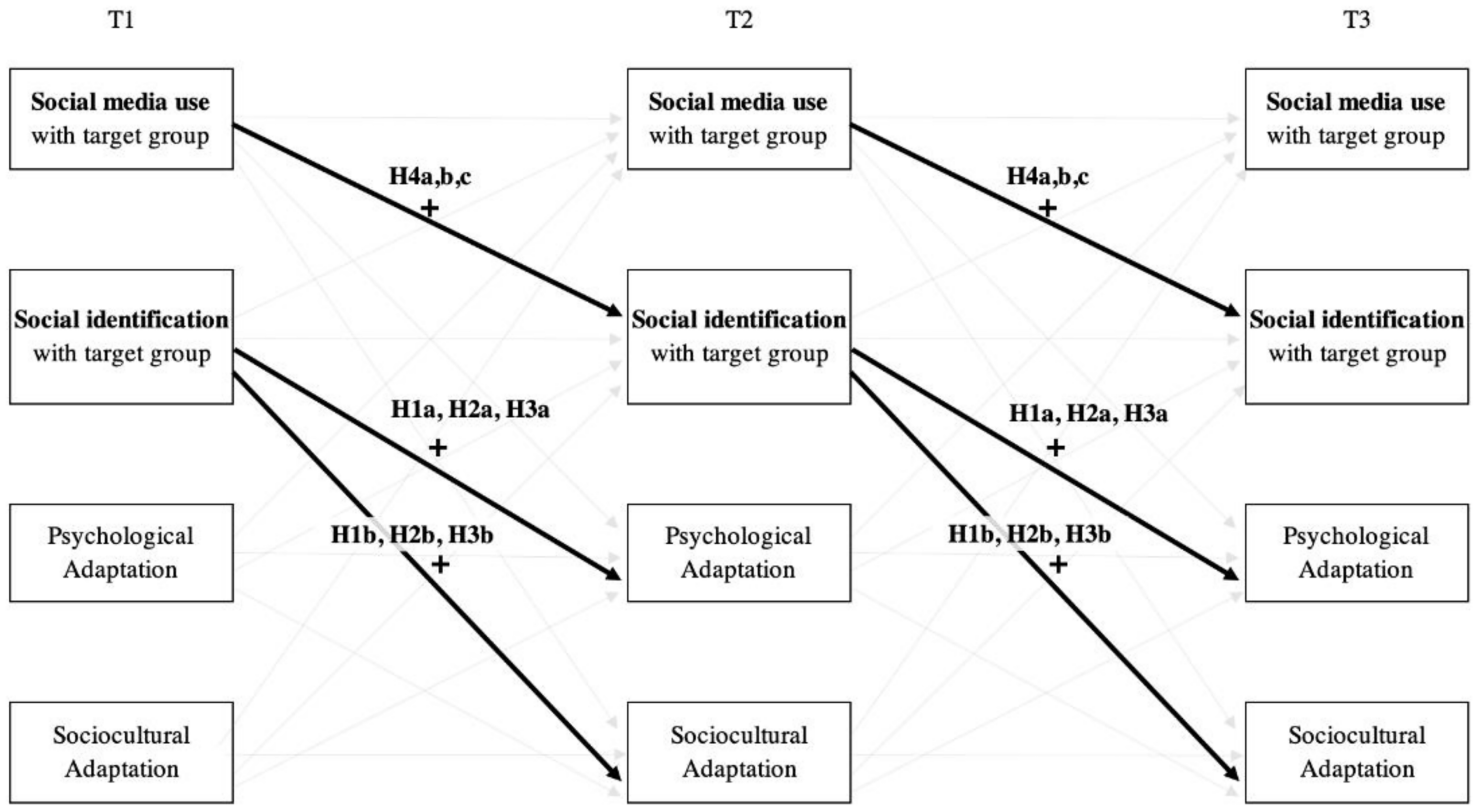
Conceptual model of the effects of social media use and social identification variables on the psychological and sociocultural adaptation of international students during their sojourn.

Although acculturation research has supported the relationship between social media and social identification, its dynamic, longitudinal and differential effects on adaptation considering multicultural groups in the host country remain understudied (Yu et al., 2019; Pang, 2020). Thus, in an effort to acknowledge the changing and dynamic nature of acculturation and adaptation (Sam and Ward, 2021), and identify predictors that lead towards a successful cross-cultural adaptation (Ward and Szabó, 2019), we analyzed the long-term effects of social media use. In the current study, we positioned social media use with the three target groups as a predictor of cross-cultural adaptation through time, by increasing the identification that international students develop towards those groups. Thus, we expected that social media use would have a positive effect on social identification with each of the target groups of local home nationals (H4a), host nationals (H4b), and international students (H4c) over time. To summarize the hypotheses outlined in the present study, a general conceptual model is provided in Figure 1.

## Materials and methods

### Sample and procedure

We tested our hypotheses in a quantitative longitudinal study. To determine the sample size, we ran a Monte Carlo power analysis using the R package *bmem* (Zhang, 2014) for a cross-lagged power model with four variables measured at three times, assuming stability coefficients of *β* = 0.60 for all auto-regressive paths (i.e., a variable at T1/T2 predicting the same variable at T2/T3) and small lagged effects (*β* = 0.20) for all lagged paths (i.e., a variable at T1/T2 predicting a different variable at T2/T3). This analysis indicated that 250 participants were needed to detect a lagged effect of this size with a power of 0.76 in a model with no equality constraints, and 200 participants in a model with equality constraints (see the Results section for more details).

Data were collected using a self-reported quantitative survey at three different time points (T1, T2, and T3) with the same group of participants. Our sample consisted of exchange students (mainly from the Erasmus program) or foreign students enrolled in an international program in different universities in Spain, Portugal and Poland. These universities were chosen because we had access to study programs in these countries that facilitated contact with participants. Most participants (95.0%) were humanities and social science students recruited through e-mail with the support of an international or exchange student coordinator at the receiving institution. The remaining 5.0% were recruited online by distributing a personalized link in different online communities. No compensation was offered to participants. All procedures, as described in this paper, were approved by the ethics review board of the first author’s institution. It is important to note that all data were collected before the start of the global COVID-19 pandemic, so the results of the study were not affected by this event.

The first wave of the study (T1) was collected at the beginning of the Fall semester throughout October and early November 2018. The second wave (T2) was collected throughout December. Finally, the third wave (T3) was collected at the end of the semester during the month of February of the following year. 320 participants completed at least one of the four scales in the questionnaire in at least one time point of the study (N_T1_ = 191, N_T2_ = 157, N_T3_ = 121; note that new participants were contacted at T2, hence total N is higher than N at T1). For this study, we only retained participants that completed the full questionnaire in at least one wave and accounted for the missing data in other waves (see Missing data section for further explanation). Thus, the final sample consisted of 233 (N_T1_ = 132, N_T2_ = 118, N_T3_ = 108).

The mean age of the participants was 22.19 years (*SD* = 3.19, Range = 18–46), 70.4% were females; 71.7% were studying in Spain, 21.0% in Portugal, and 6.0% in Poland. In total, 35 nationalities were represented in the sample (see figures in the Supplementary materials). The majority of participants were from a European country (84.1%), such as Italy (26.6%), France (18.5%), Germany (13.3%), and United Kingdom (6.4%). From other continents, the most represented countries were Brazil (2.1%), United States (1.7%), Japan (1.3%), and Australia (0.9%); 5.2% did not report their nationality.

### Measures

*Social Media Use* was measured with a 9-item scale by asking respondents about their frequency of social media use (Rosen et al., 2013; Sheldon and Bryant, 2016). Items included ‘How often do you interact with profiles (tagging, posting, sharing, etc.)’ and ‘How often do you private or group text chat’. A Likert scale ranging from 1 (Never) to 7 (Very often) was provided. The scale was presented three times to evaluate social media use with the three target groups: local home nationals (α_T1_ = 0.92, α_T2_ = 0.94, α_T3_ = 0.92), host nationals (α_T1_ = 0.91, α_T2_ = 0.89, α_T3_ = 0.90), and international students (α_T1_ = 0.87, α_T2_ = 0.89, α_T3_ = 0.92).

*Social Identification* was operationalized using a 6-item scale, adapted from Mael and Tetrick (1992), that measures identification with a psychological group based on shared experiences and characteristics. Items included ‘When someone criticizes [target group], it feels like a personal insult’ and ‘When I talk about [target group], I usually say “we,” rather than “they”’. A Likert scale ranging from 1 (Strongly disagree) to 7 (Strongly agree) was presented. The participants responded to the scale three times to address the identification they had with the three target groups: local home nationals (α_T1_ = 0.75, α_T2_ = 0.75, α_T3_ = 0.81), host nationals (α_T1_ = 0.82, α_T2_ = 0.79, α_T3_ = 0.85), and international students (α_T1_ = 0.85, α_T2_ = 0.83, α_T3_ = 0.85).

*Psychological Adaptation* was measured with the complete 10-item scale by Demes and Geeraert (2014). Sample items included ‘Excited about being in the host country’ and ‘Sad to be away from your home country’. Items were presented with a Likert scale ranging from 1 (Never) to 7 (Always), negative items were reverse-coded prior to analysis to match a better adaptation with a higher score (α_T1_ = 0.83, α_T2_ = 0.83, α_T3_ = 0.84).

Finally, *Sociocultural Adaptation* was measured with nine items from the revised scale (SCAS-R) by Wilson et al. (2017), which measures cultural competencies in the host country. Items included ‘Building and maintaining relationships’, ‘Finding my way around’, and ‘Interacting at social events’ (α_T1_ = 0.78, α_T2_ = 0.83, α_T3_ = 0.86). A Likert scale was presented ranging from 1 (Not at all competent) to 7 (Extremely competent).

### Missing data

To check for the missingness mechanism, we compared, in a series of t-tests, participants who finished all three waves of the study with those who completed one or two waves. Completers did not differ significantly from dropouts on any of the focal variables of this study (all *p*s > 0.05, e.g.: psychological adaptation, *t*(131) = −1.36, *p* = 0.18; sociocultural adaptation, *t*(130) = 0.10, *p* = 0.925; identification with host nationals *t*(132) = −1.80, *p* = 0.07, social media use with host nationals *t*(132) = −1.13, *p* = 0.26). This pattern was consistent with the missing at random (MAR) mechanism (Rubin, 1976; see also Graham, 2009 for an overview of missing data mechanisms). We used full information maximum likelihood to account for missing data (viable technique under MAR; Schafer and Graham, 2002; Graham, 2009), hence all results refer to *N* = 233.

## Results

### Model construction

For the current study, we specified three cross-lagged panel models (CLPM), one for each target group (local home nationals, host nationals, internationals). Each model included T1, T2, and T3 scores on psychological adaptation, sociocultural adaptation, social media use with one of the target groups, and social identification with the same group. Cross-lagged panel models are used to estimate cross-lagged effects of one variable at an earlier time on another variable at a later time through the analysis of residual change, controlling for earlier levels of the outcome variable; thus, allowing to establish temporal precedence between variables (Selig and Little, 2012; Kearney, 2017). However, results need to be interpreted with caution since they are time-interval dependent (Kuiper and Ryan, 2018), and the nature of the variables studied does not allow for causal inference solely from statistical models (Selig and Little, 2012). All analyses were conducted in the R package *lavaan* 0.6–5 (Rosseel, 2012). In all cases, we accounted for non-normal distributions of the four variables (all Kolmogorov–Smirnov *p*s < 0.001) by using robust maximum likelihood (MLR) estimation with Yuan-Bentler scaled χ^2^ test statistics and Huber-White robust standard errors.

We regressed the T2 score of each variable on its own T1 value and the T1 values of the remaining variables, and the T3 score of each variable on its own T2 value and the T2 values of the remaining variables. A conservative test of the causal order of variables requires the lagged effects to be the same between all timepoints (Mund and Nestler, 2019). Therefore, we constrained all auto-regressive paths, all lagged paths between variables and all within-wave residual correlations between variables to equality between all timepoints. These constrained models showed a close fit to the data for local home nationals (Model 1), χ^2^(38) = 56.603, *p* = 0.027, CFI = 0.950; TLI = 0.921; SRMR = 0.072; RMSEA = 0.045, 90% CI [0.016, 0.068], *p*_close_ = 0.582; for host nationals (Model 2), χ^2^(38) = 47.956, *p* = 0.129, CFI = 0.970; TLI = 0.952; SRMR = 0.073; RMSEA = 0.032, 90% CI [0.000, 0.058], *p*_close_ = 0.819; and for internationals (Model 3), χ^2^(38) = 45.069, *p* = 0.200, CFI = 0.978; TLI = 0.966; SRMR = 0.057; RMSEA = 0.029, 90% CI [0.000, 0.059], *p*_close_ = 0.897.

To ensure that the constrained models were the optimal representation of the data, we additionally tested unconstrained models (i.e., models with all paths estimated freely except for stability coefficients) and we compared them to the fully constrained models. Setting all paths free did *not* improve model fit significantly for local home nationals (Model 1), ∆χ^2^(18) = 27.174, *p* = 0.076; for host country nationals (Model 2), ∆χ^2^(18) = 21.839, *p* = 0.239; and for internationals (Model 3), ∆χ^2^(18) = 17.531, *p* = 0.487. Therefore, we retained the fully constrained models for all three target groups.

### Hypothesis testing

Below, we present the results per target group and focus only on the significant lagged effects found in the three models outlined. A full overview of descriptive statistics (Supplementary Table S1) cross-lagged estimates (Supplementary Tables S2–S4) and covariance estimates (Supplementary Tables S5–S7) can be found in the Supplementary materials. For each group we first describe results according to the effects of identification on cross-cultural adaptation (H1, H2, or H3), and then describe findings related to the effect of social media on identification (H4).

### Identification and social media use with local home nationals

**Figure 2:**
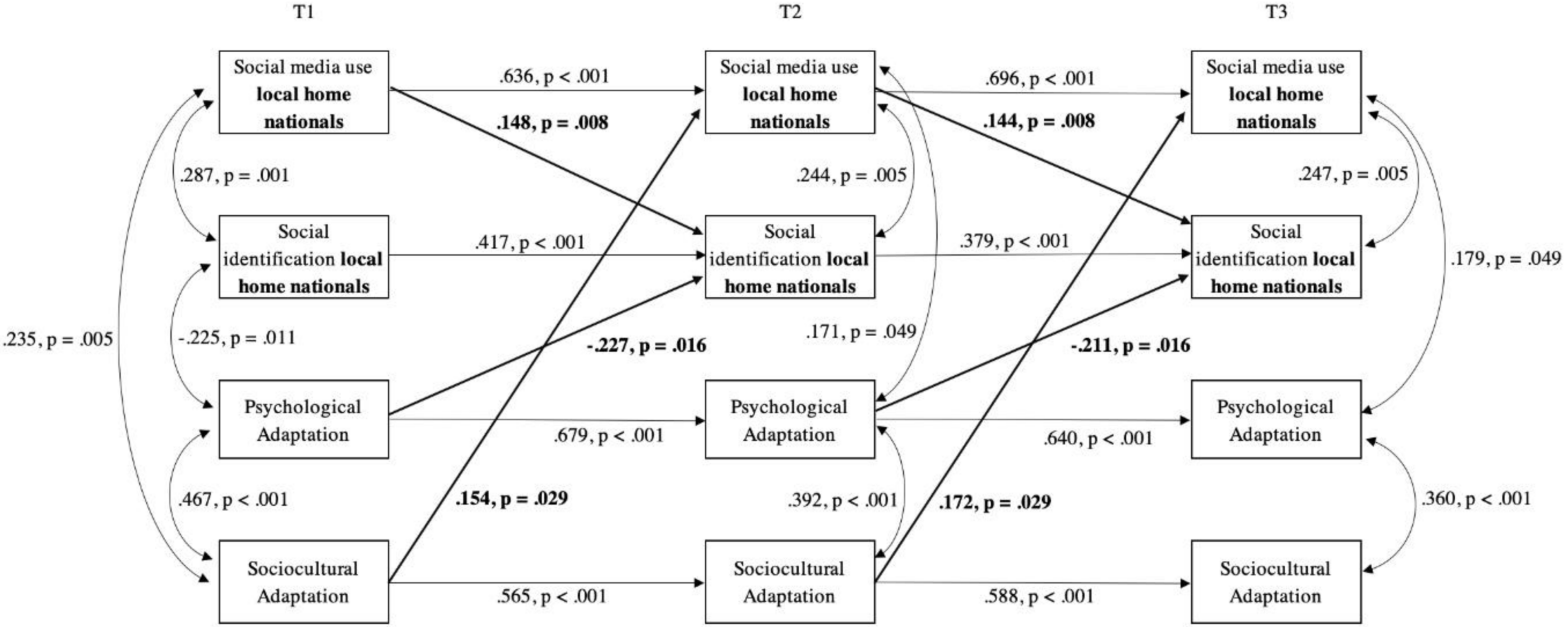
Cross-lagged panel model of the relationships between social media use and social identification with local home nationals, and psychological and sociocultural adaptation of international students. Only significant paths are shown. Results have been standardized and constrained to equality between time points. Model Fit: χ^2^(38) = 56.603, *p* = 0.027, CFI = 0.950; TLI = 0.921; SRMR = 0.072; RMSEA = 0.045, 90% CI [0.016, 0.068], *p*_close_ = 0.582; *N* = 233.

Hypothesis 1 predicted a positive effect of identification with local home nationals on both psychological (a) and sociocultural adaptation (b) of international students; Hypothesis 4a predicted a positive effect of social media use targeted at local home nationals on the identification with that group. Our findings showed no lagged effect of identification on either dimension of adaptation, which prompted us to reject Hypothesis 1a and 1b. However, social media use targeted at this group at an earlier wave was positively associated with identification with this group at a later wave, showing support for Hypothesis 4a. Unexpectedly, sociocultural adaptation at an earlier wave was positively associated with social media use targeted at local home nationals at a later wave, while psychological adaptation at an earlier wave was negatively associated with identification with local home nationals at a later wave (see Figure 2).

### Identification and social media use with host nationals

**Figure 3:**
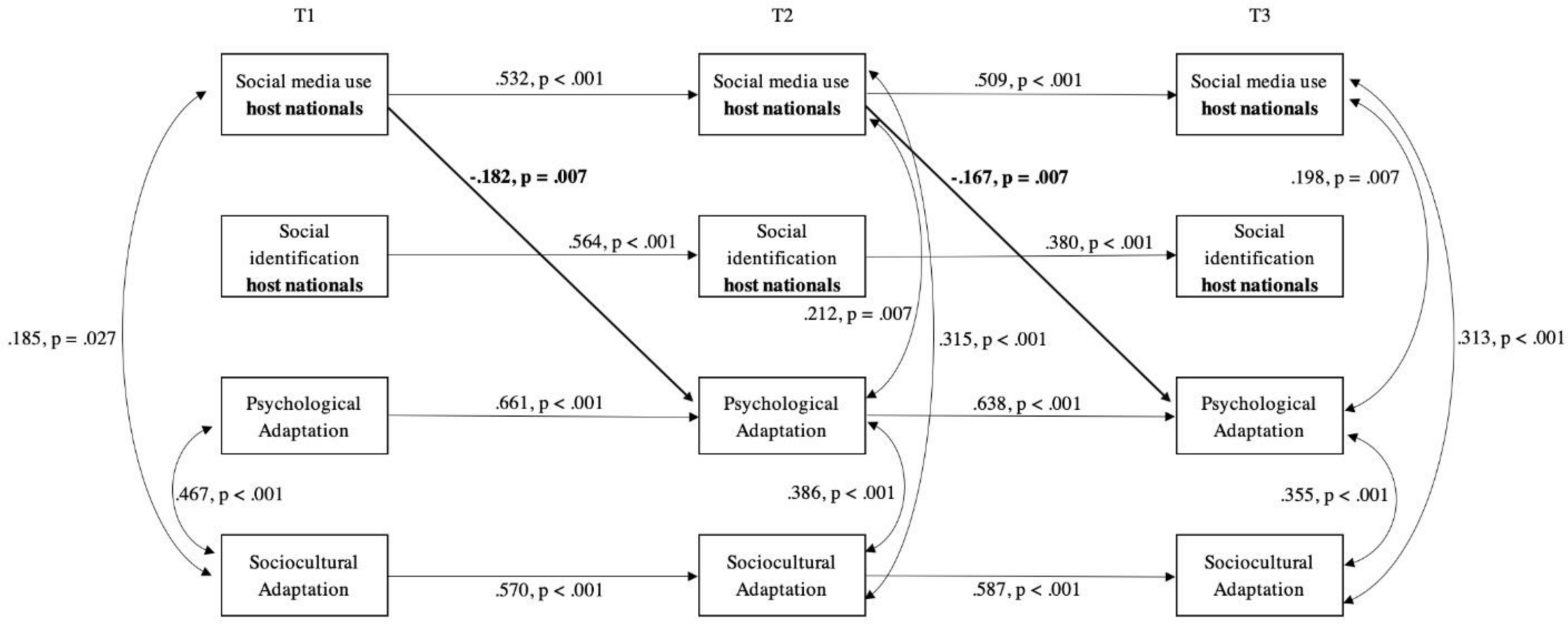
Cross-lagged panel model of the relationships between social media use and social identification with host nationals, and psychological and sociocultural adaptation of international students. Only significant paths are shown. Results have been standardized and constrained to equality between time points. Model Fit: χ^2^(38) = 47.956, *p* = 0.129, CFI = 0.970; TLI = 0.952; SRMR = 0.073; RMSEA = 0.032, 90% CI [0.000, 0.058], *p*_close_ = 0.819; *N* = 233.

In the case of host nationals, Hypothesis 2 predicted a positive effect of identification with this group on both psychological (a) and sociocultural adaptation (b) of international students; Hypothesis 4b, predicted a positive effect of social media use targeted at host nationals on the identification with that group. Contrary to our expectations, no lagged effect was found of identification with host nationals on either dimension of adaptation; similarly, social media use targeted at this group had no lagged effects on identification. Therefore, Hypotheses 2a, 2b, and 4b were not supported. Interestingly, social media use targeted at host nationals at an earlier wave was negatively associated with psychological adaptation and had no lagged effect on sociocultural adaptation (see Figure 3).

### Identification and social media use with other international students

**Figure 4:**
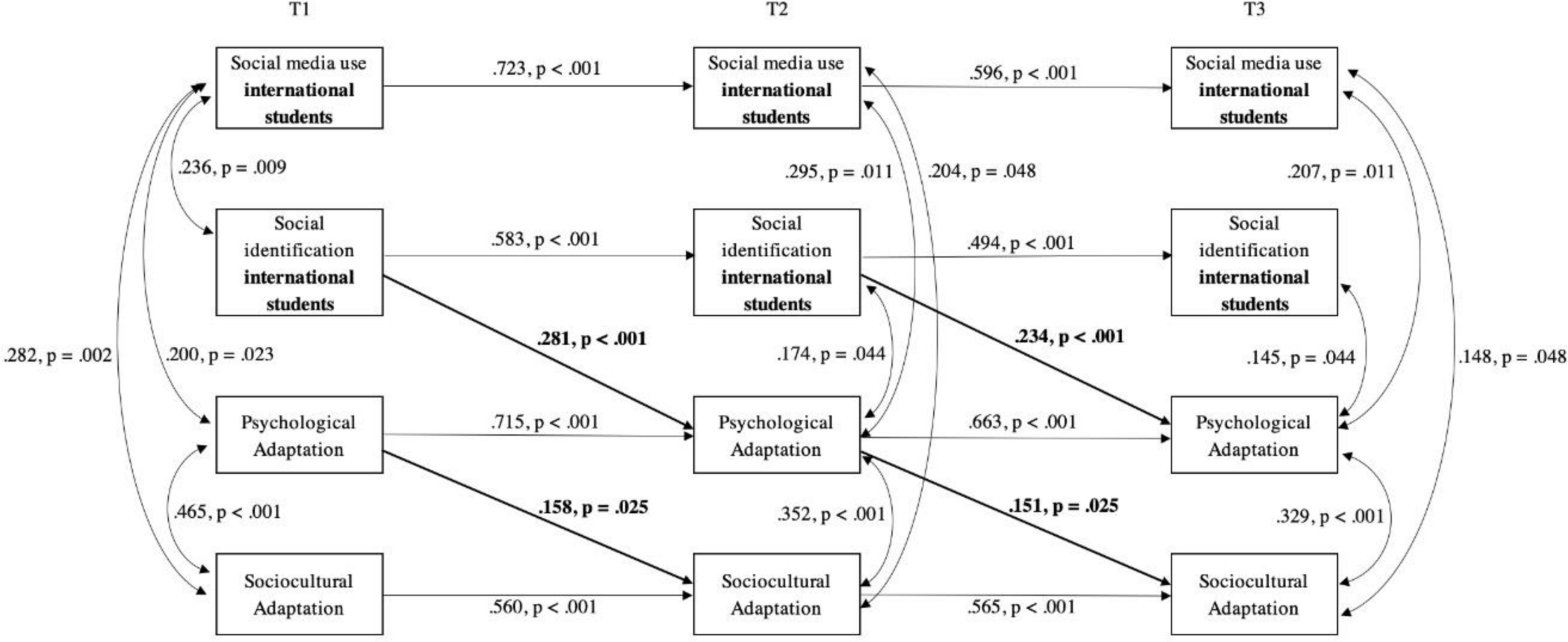
Cross-lagged panel model with three time points of the relationships between social media use and social identification with other international students, and psychological and sociocultural adaptation of international students. Only significant paths are shown. Results have been standardized and constrained to equality between time points. Model Fit: χ^2^(38) = 45.069, *p* = 0.200, CFI = 0.978; TLI = 0.966; SRMR = 0.057; RMSEA = 0.029, 90% CI [0.000, 0.059], *p*_close_ = 0.897; *N* = 233.

In the case of other international students, Hypothesis 3 predicted that social identification with this group would have a positive effect on both psychological (a) and sociocultural adaptation (b); Hypothesis 4c predicted a positive effect of social media use targeted at international students on the identification with that group. The analysis showed that identification with the group of international students at an earlier wave was positively associated with psychological adaptation at a later wave, but not with sociocultural adaptation. Thus, Hypothesis 3a was supported while Hypothesis 3b was rejected. Opposite to our expectations, social media use targeted at international students did not have lagged effects on identification, which prompted us to reject Hypothesis 4c. Finally, psychological adaptation at an earlier wave was positively related to sociocultural adaptation at a later wave (see Figure 4).

## Discussion

### Findings and theoretical implications

Previous research has identified the importance of social media in the acculturation process of international students, highlighting possible benefits that range from creating and maintaining social ties, to fostering identification with the groups they interact with (Cemalcilar et al., 2005; Park and Noh, 2018; Hofhuis et al., 2019). The results of this study, however, suggest that those benefits may depend on the groups international students interact with. Furthermore, the current research also responds to the call for more longitudinal research, that allows for a better understanding of the acculturation process across time (Ward and Geeraert, 2016; Ward and Szabó, 2019) and contributes to the discussion of theoretical relationships that are mostly based on cross-sectional research (Bierwiaczonek and Kunst, 2021; Kunst, 2021). In a cross-lagged model, we tested the longitudinal effects of social media use on social identification of international students with three target groups: (1) local home nationals, (2) host nationals, and (3) other international students. We also examined the longitudinal effect of identification with each of these groups on the psychological and sociocultural adaptation of international students. Below we discuss our results and their theoretical implications separately per target group.

### Local home nationals

Poorer psychological adaptation predicted greater identification with the group of local home nationals. Contrary to our expectations, these results suggest that the negative association between identification with co-nationals (living in the home country) and psychological adaptation found in previous research (e.g., Lim and Pham, 2016; Hofhuis et al., 2019; Gibbs et al., 2020), might hold true for the group of local home nationals as well. Differently than previous cross-sectional studies, which place adaptation as an outcome, our findings suggest that this association may reflect a response to struggling with psychological adaptation. Similarly to Pekerti et al. (2020), we argue that poor psychological adaptation, reflecting a struggle with the stressful aspects of intercultural transitions, may lead international students to seek their national in-group as a coping mechanism. This group, perhaps the most salient and obvious in-group (Cachia and Maya-Jariego, 2018), is likely more available and easier to connect with than other groups present in the host country context; thus, students are likely to turn to the local home national in-group to fulfill their need for connectedness and belonging (Ward et al., 2001; Collins, 2010), which might not be completely fulfilled by their social networks back home (e.g., Nguyen et al., 2021).

As we expected, social media targeted at the group of local home nationals had a positive lagged effect on the identification with this group. This finding goes in line with previous studies that have explored this relationship. For example, consuming social media related to local home nationals may reinforce the identification with this group by exchanging information that can only be interpreted with shared meaning, such as migration background or local language (e.g., C. Li and Tsai, 2015). Social media use with local home nationals may represent a way to buffer acculturative stress related to the loss of networks from the home country and provide bonding experiences in the host country context (Sandel, 2014) that in turn increase the identification with this group. Furthermore, social media targeted at local home nationals may be perceived as trusted channels of information that reinforce home cultural values that are central to international students’ identity (Vuong and Napier, 2015).

Although we did not contemplate a direct association between social media and adaptation variables in our hypotheses, previous literature showed that social ties and social media contact with local home nationals may hinder sociocultural adaptation in the long term (e.g., Geeraert et al., 2014; Rui and Wang, 2015). Surprisingly, our findings suggest that this might not be the only scenario. Specifically, in our sample, better sociocultural adaptation of international students preceded an increase in social media use targeted at local home nationals, suggesting that international students first needed to navigate and adapt in their host society in order to connect with their local national in-group. These results can be best understood when keeping in mind that sociocultural adaptation implies successfully navigating a new cultural milieu (Ward and Szabó, 2019) which can help in accessing different social ties (e.g., Sakurai et al., 2010), including local home nationals.

### Host nationals

Our main result regarding the group of host nationals is that social media use targeted at this group predicted poorer psychological adaptation of international students; no other lagged effects were found, which points to the possibility that host nationals might be the least accessible identity group (e.g., Ward et al., 2001; Ramos et al., 2016). Our findings reveal that, while social media use with host nationals may help reduce acculturative stress in some instances (e.g., Park et al., 2014; Cao et al., 2018; Li and Peng, 2019; Billedo et al., 2020; Pang, 2020), it may also hinder sojourner’s well-being in a longer term. On the one hand, the information accessed through social media use with host nationals might not correspond with the core values of international students, preventing them from practicing values that might help their adaptation (Vuong and Napier, 2015). On the other hand, the findings might reflect the challenges faced by international students when using social media targeted at host nationals. For example, attempts to connect with host nationals *via* social media may trigger feelings of disconnect from the host national group, a decrease of perceived support from that group (e.g., Billedo et al., 2019), or simply result in confusion and misunderstandings due to language barriers. In other words, international students might experience greater challenges when trying to connect with host nationals (Szabó et al., 2020), negatively affecting their well-being and psychological adaptation (e.g., Bethel et al., 2016; Taušová et al., 2019).

### International students

Social identification with other international students predicted better psychological adaptation, and this, in turn, predicted better sociocultural adaptation; no lagged effects were found related to social media use targeted at this group. Our findings point to the importance of the international student group as one main resource to achieve cross-cultural adaptation. We argue that proximity and the sharing of experiences might allow for the benefits of social identification to take place during the acculturation experience (Bierwiaczonek et al., 2017).

These findings go in line with previous research that emphasizes the importance of bonding experiences and friendships among the international student group during the sojourn (Hendrickson et al., 2011; Beech, 2018; Quinton, 2020). International students share an experience that transcends different areas of their lives; they are in a new host country, move around similar contexts within and outside university walls and face similar academic and acculturating challenges. Thus, students may be motivated to actively look for the group of international students, as they represent a more immediate source of support (e.g., Beech, 2018; Pekerti et al., 2020; Shu et al., 2020), and enhance the feeling of belonging (Kashima and Loh, 2006; Gomes et al., 2014; Schartner, 2015; Bierwiaczonek et al., 2017; Taušová et al., 2019).

In line with Taušová et al. (2019), we believe that a comprehensive model of acculturation needs to consider the role of fellow sojourners for individual outcomes, as this appears to be one important group to share the experience of adaptation with. This is especially important in the case of international students, who usually embark on short-term mobility and move around in spaces that enhance the contact with other internationals (e.g., Fincher and Shaw, 2011; Williams and Johnson, 2011; Hendrickson, 2018); which may influence how open to other cultures they become (Vuong and Napier, 2015) and their future life choices (Nada and Legutko, 2022).

In this context, the lack of lagged effects of social media use targeted at other international students may be surprising. In our view, this lack may suggests that social media alone are not a sufficient means to significantly support the acculturation process (e.g., Damian and Van Ingen, 2014; Rui and Wang, 2015) or the process of building identification with other international students, and in-person experiences might actually be the decisive component. Therefore, future research could compare face to face contact and social media contact, to identify under what conditions social media may play a positive role in acculturation and cross-cultural adaptation of international students.

## Limitations and future research

As any empirical study, this research bears some limitations. We observed a high dropout rate, however, this is common struggle of longitudinal research, and we accounted for it in the best way possible by using full information maximum likelihood. Another limitation of the present work is that data collection was based on self-reports. The scales in the study have been widely used in the field, which allows us to directly compare our findings to previous work. However, future research should also consider using divergent measures, such as behavioral data, when studying variables related to intercultural skills and adaptation (Ward, 2022).

Considering this study’s longitudinal nature and the length of the questionnaire, several relevant variables describing social media use had to be omitted. For example, previous studies have found that the way sojourners communicate with different cultural groups is influenced by media richness (e.g., Lee and Katz, 2015; McKelvy and Chatterjee, 2017), and that the benefits of social media use for cross-cultural adaptation may depend on the individual’s active or passive social media use (Pang, 2020). Furthermore, social media purposes and specific social media platforms can impact differently the acculturation process (e.g., Cao et al., 2018; Hu et al., 2018, 2020). Thus, future research could longitudinally test these and other aspects of social media use for international students’ adaptation.

## Practical implications

Our findings exemplify the importance of addressing the multicultural groups that are part of international students’ lives while abroad. Although the feeling of belonging to a group in the host country is of utmost importance for the well-being of international students, it seems that the benefits might arise when the group shares similar experiences. Therefore, practitioners and university staff involved in the process of student mobility should promote meaningful relationships among international students as they can be one important source of support. Furthermore, as our results show that social media use can have a negative impact on students’ well-being, we encourage university staff to promote a critical use of social media among the population of international students, especially when these media are targeted at host nationals. Recognizing that different groups can have different benefits or disadvantages might aid the cross-cultural adaptation process of international students.

## Conclusion

The main aim of this longitudinal research was to explore how the different groups that are available to international students within the host country impact their cross-cultural adaptation, namely through social identification processes and social media use targeted at these groups. In sum, our findings show that identification processes with groups that share similar experiences are vital for the adaptation of international students, and that the benefits of social media during the acculturation process may depend on the different groups that are targeted through these channels. However, more longitudinal research on cross-cultural adaptation is needed to fully understand this dynamic process and the role that social media, a key ingredient of the contemporary study abroad experience, play in the adaptation and acculturation of international students.

## Data availability statement

The anonymized data supporting the conclusions of this article will be made available by the authors, without undue reservation.

## Ethics statement

The studies involving human participants were reviewed and approved by ESHCC Ethics Review Committee, Erasmus University Rotterdam. The patients/participants provided their written informed consent to participate in this study.

## Author contributions

LG-A collected part of the data, performed the statistical analyses, and wrote the paper. JH collected part of the data and co-wrote the paper. KB performed the statistical analyses and provided essential revisions to the paper. CC collected part of the data and provided essential revisions to the paper. All authors contributed to the article and approved the submitted version.

## Funding

The APC for this publication is supported by the University of Oslo. The data collection described in this paper was supported by a scholarship from the European Commission through the European Master in the Psychology of Global Mobility, Inclusion and Diversity in Society (G-MINDS) awarded to the first author.

## Conflict of interest

The authors declare that the research was conducted in the absence of any commercial or financial relationships that could be construed as a potential conflict of interest.

## Publisher’s note

All claims expressed in this article are solely those of the authors and do not necessarily represent those of their affiliated organizations, or those of the publisher, the editors and the reviewers. Any product that may be evaluated in this article, or claim that may be made by its manufacturer, is not guaranteed or endorsed by the publisher.

## Supplementary material

The Supplementary material for this article can be found online at: https://www.frontiersin.org/articles/10.3389/fpsyg.2022.1013375/full#supplementary-material
